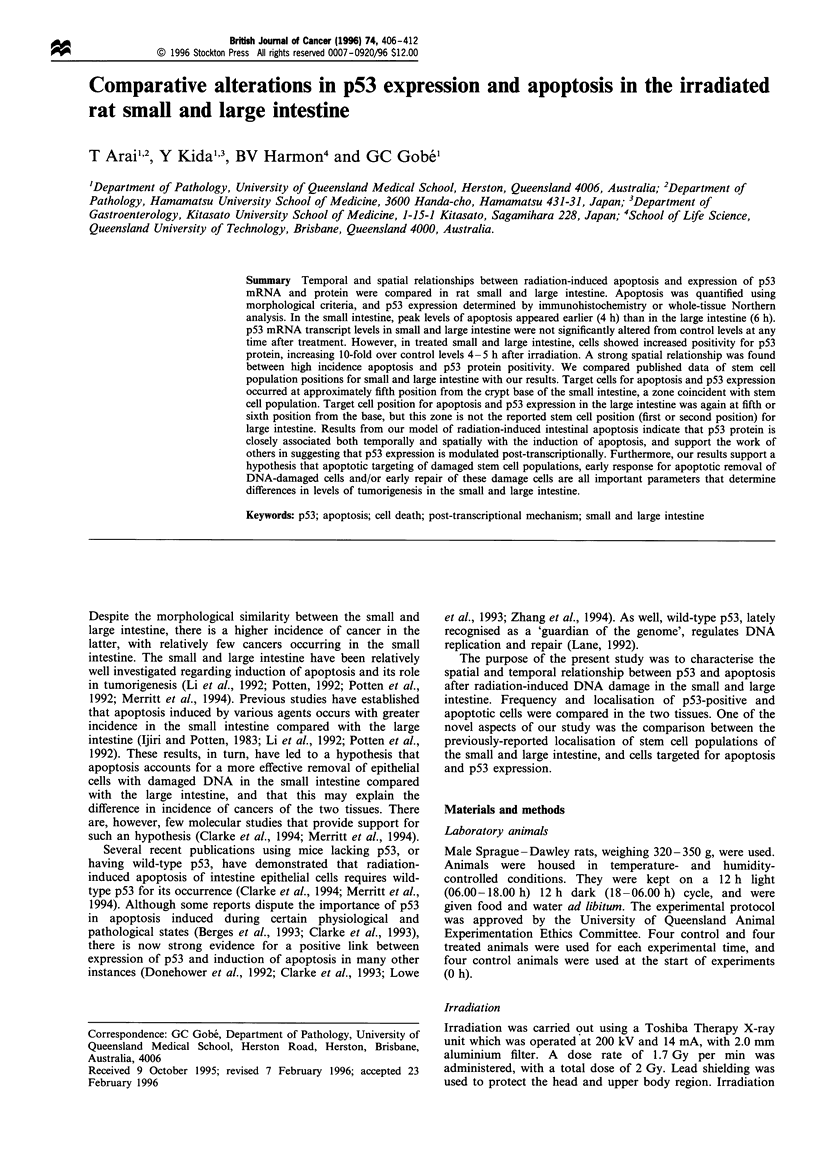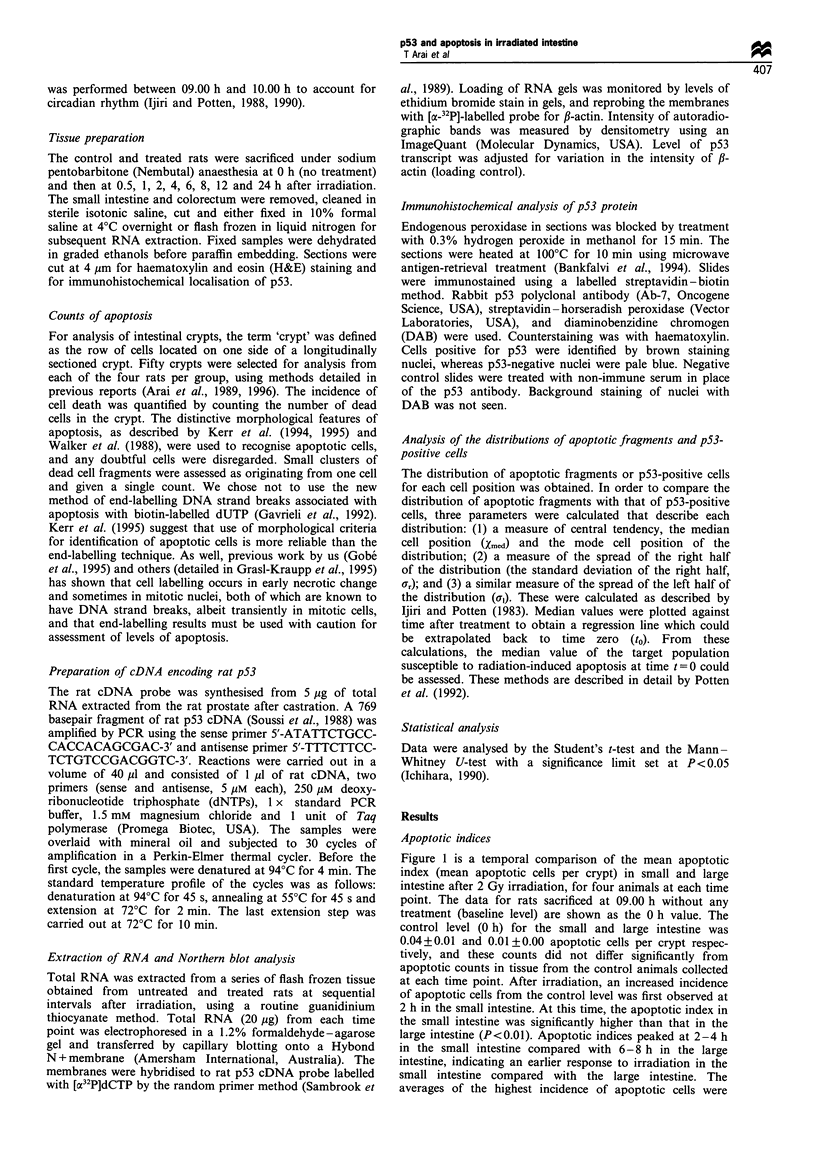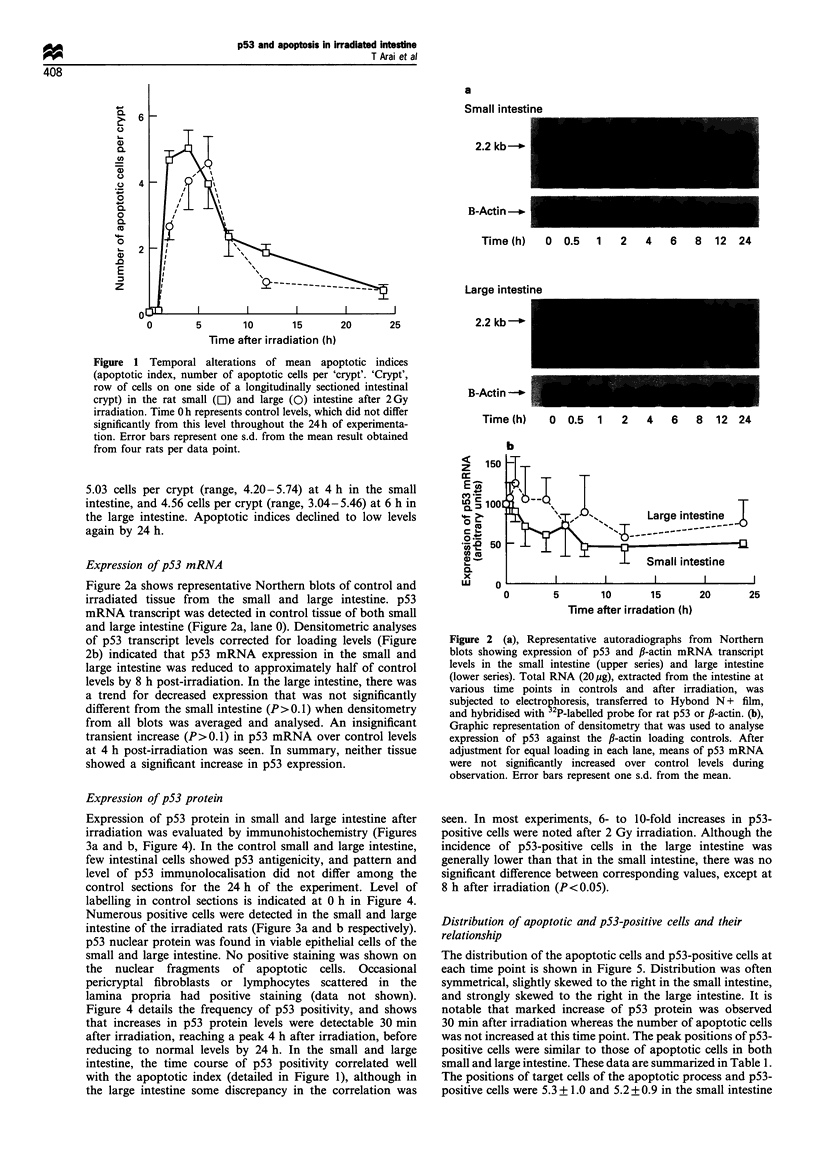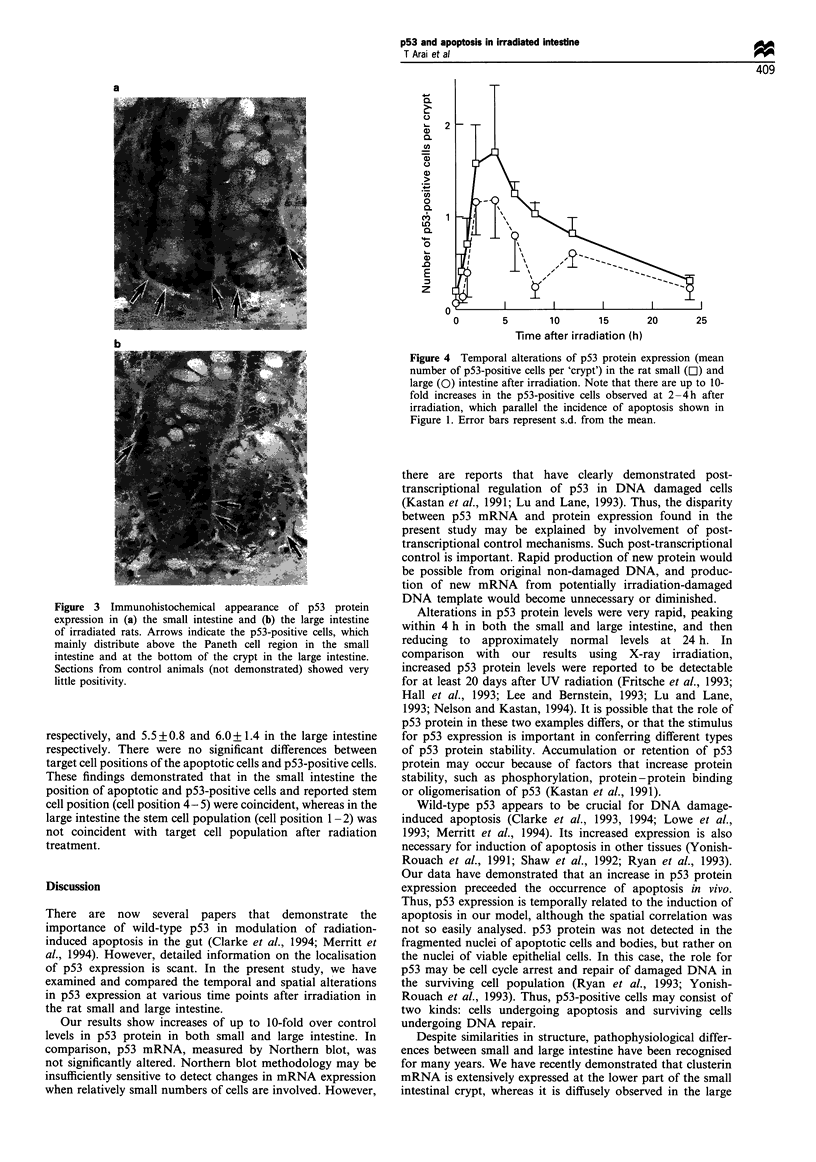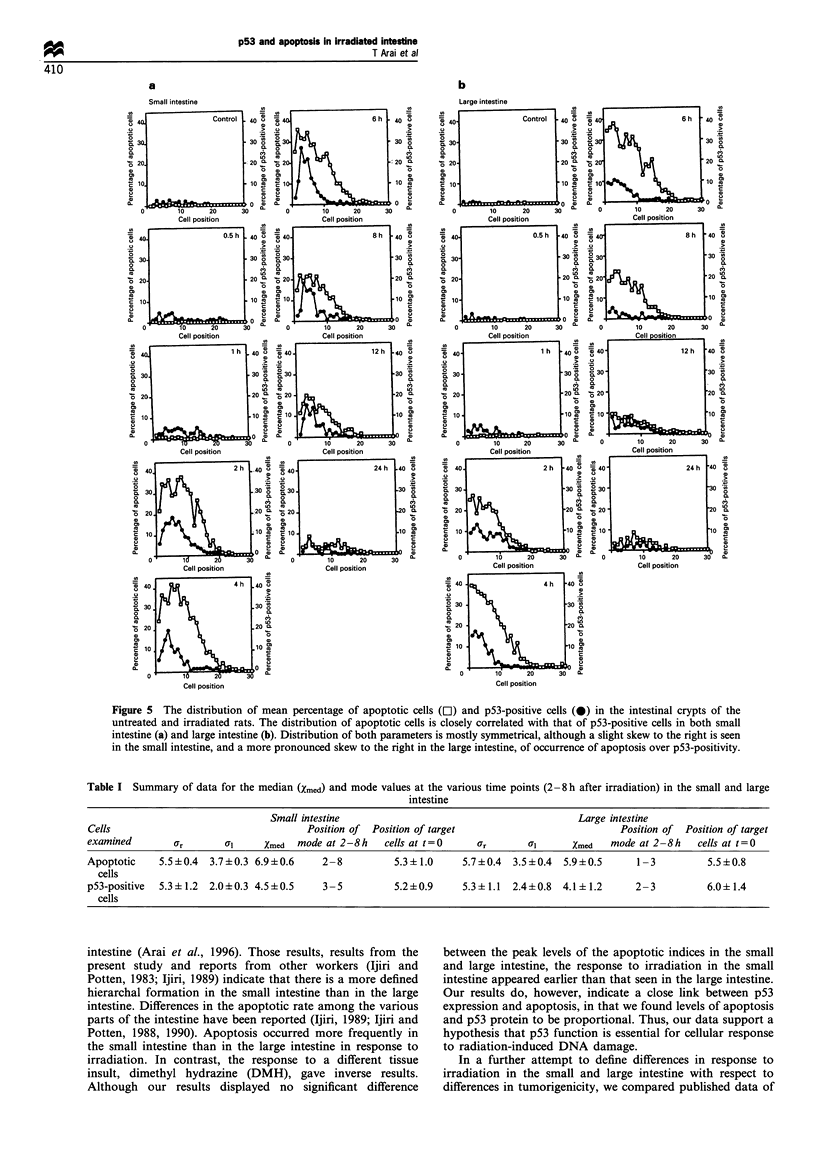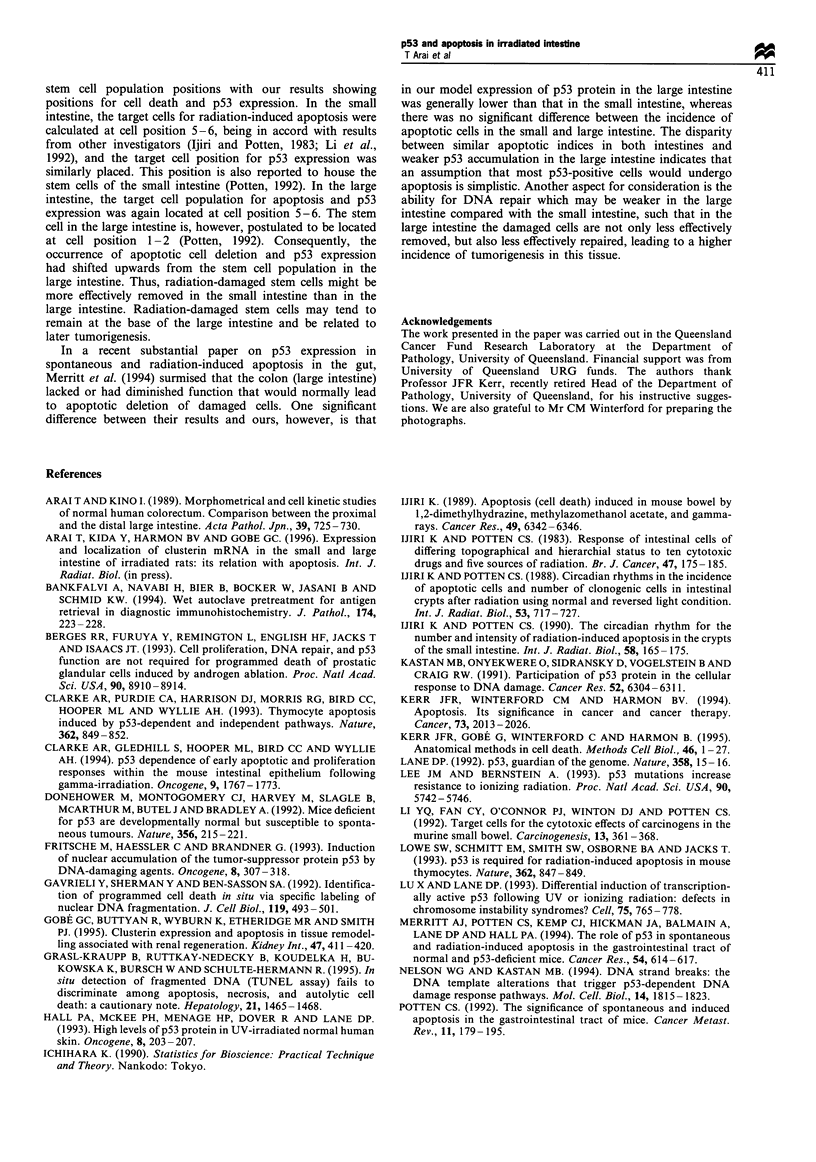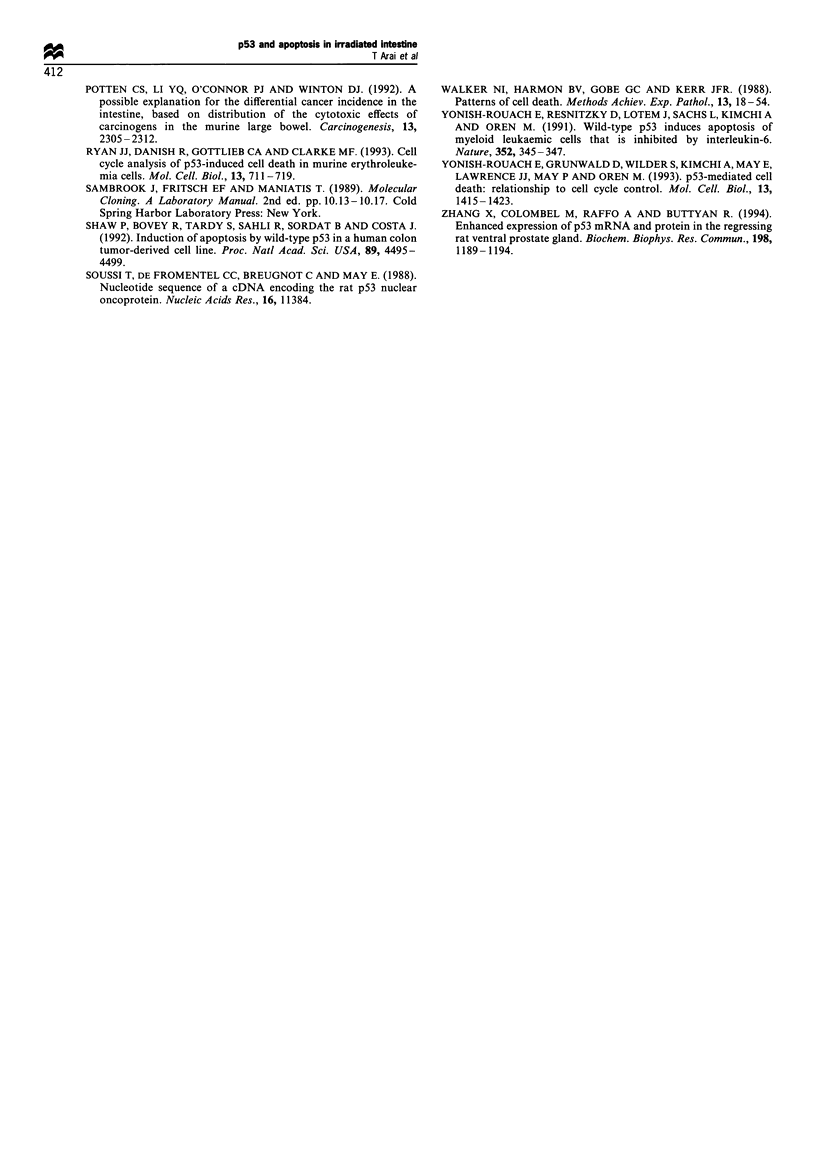# Comparative alterations in p53 expression and apoptosis in the irradiated rat small and large intestine.

**DOI:** 10.1038/bjc.1996.373

**Published:** 1996-08

**Authors:** T. Arai, Y. Kida, B. V. Harmon, G. C. Gobé

**Affiliations:** Department of Pathology, University of Queensland Medical School, Herston, Australia.

## Abstract

**Images:**


British Journal of Cancer (1996) 74, 406-412
© 1996 Stockton Press All rights reserved 0007-0920/96 $12.00

### Comparative alterations in p53 expression and apoptosis in the irradiated rat small and large intestine

T Arai[1,2], Y Kida[1,3], BV Harmon[4] and GC Gobé[1]

[1]Department of Pathology, University of Queensland Medical School, Herston, Queensland 4006, Australia; [2]Department of Pathology, Hamamatsu University School of Medicine, 3600 Handa-cho, Hamamatsu 431-31, Japan; [3]Department of Gastroenterology, Kitasato University School of Medicine, 1-15-1 Kitasato, Sagamihara 228, Japan; [4]School of Life Science, Queensland University of Technology, Brisbane, Queensland 4000, Australia.

**Summary** Temporal and spatial relationships between radiation-induced apoptosis and expression of p53 mRNA and protein were compared in rat small and large intestine. Apoptosis was quantified using morphological criteria, and p53 expression determined by immunohistochemistry or whole-tissue Northern analysis. In the small intestine, peak levels of apoptosis appeared earlier (4 h) than in the large intestine (6 h). p53 mRNA transcript levels in small and large intestine were not significantly altered from control levels at any time after treatment. However, in treated small and large intestine, cells showed increased positivity for p53 protein, increasing 10-fold over control levels 4–5 h after irradiation. A strong spatial relationship was found between high incidence apoptosis and p53 protein positivity. We compared published data of stem cell population positions for small and large intestine with our results. Target cells for apoptosis and p53 expression occurred at approximately fifth position from the crypt base of the small intestine, a zone coincident with stem cell population. Target cell position for apoptosis and p53 expression in the large intestine was again at fifth or sixth position from the base, but this zone is not the reported stem cell position (first or second position) for large intestine. Results from our model of radiation-induced intestinal apoptosis indicate that p53 protein is closely associated both temporally and spatially with the induction of apoptosis, and support the work of others in suggesting that p53 expression is modulated post-transcriptionally. Furthermore, our results support a hypothesis that apoptotic targeting of damaged stem cell populations, early response for apoptotic removal of DNA-damaged cells and/or early repair of these damage cells are all important parameters that determine differences in levels of tumorigenesis in the small and large intestine.

**Keywords:** p53; apoptosis; cell death; post-transcriptional mechanism; small and large intestine

Despite the morphological similarity between the small and large intestine, there is a higher incidence of cancer in the latter, with relatively few cancers occurring in the small intestine. The small and large intestine have been relatively well investigated regarding induction of apoptosis and its role in tumorigenesis (Li *et al.*, 1992; Potten, 1992; Potten *et al.*, 1992; Merritt *et al.*, 1994). Previous studies have established that apoptosis induced by various agents occurs with greater incidence in the small intestine compared with the large intestine (Ijiri and Potten, 1983; Li *et al.*, 1992; Potten *et al.*, 1992). These results, in turn, have led to a hypothesis that apoptosis accounts for a more effective removal of epithelial cells with damaged DNA in the small intestine compared with the large intestine, and that this may explain the difference in incidence of cancers of the two tissues. There are, however, few molecular studies that provide support for such an hypothesis (Clarke *et al.*, 1994; Merritt *et al.*, 1994).

Several recent publications using mice lacking p53, or having wild-type p53, have demonstrated that radiation-induced apoptosis of intestine epithelial cells requires wild-type p53 for its occurrence (Clarke *et al.*, 1994; Merritt *et al.*, 1994). Although some reports dispute the importance of p53 in apoptosis induced during certain physiological and pathological states (Berges *et al.*, 1993; Clarke *et al.*, 1993), there is now strong evidence for a positive link between expression of p53 and induction of apoptosis in many other instances (Donehower *et al.*, 1992; Clarke *et al.*, 1993; Lowe *et al.*, 1993; Zhang *et al.*, 1994). As well, wild-type p53, lately recognised as a 'guardian of the genome', regulates DNA replication and repair (Lane, 1992).

The purpose of the present study was to characterise the spatial and temporal relationship between p53 and apoptosis after radiation-induced DNA damage in the small and large intestine. Frequency and localisation of p53-positive and apoptotic cells were compared in the two tissues. One of the novel aspects of our study was the comparison between the previously-reported localisation of stem cell populations of the small and large intestine, and cells targeted for apoptosis and p53 expression.

### Materials and methods

### *Laboratory animals*

Male Sprague–Dawley rats, weighing 320–350 g, were used. Animals were housed in temperature- and humidity-controlled conditions. They were kept on a 12 h light (06.00–18.00 h) 12 h dark (18–06.00 h) cycle, and were given food and water *ad libitum*. The experimental protocol was approved by the University of Queensland Animal Experimentation Ethics Committee. Four control and four treated animals were used for each experimental time, and four control animals were used at the start of experiments (0 h).

### *Irradiation*

Irradiation was carried out using a Toshiba Therapy X-ray unit which was operated at 200 kV and 14 mA, with 2.0 mm aluminium filter. A dose rate of 1.7 Gy per min was administered, with a total dose of 2 Gy. Lead shielding was used to protect the head and upper body region. Irradiation

Correspondence: GC Gobé, Department of Pathology, University of Queensland Medical School, Herston Road, Herston, Brisbane, Australia, 4006
Received 9 October 1995; revised 7 February 1996; accepted 23 February 1996

was performed between 09.00 h and 10.00 h to account for circadian rhythm (Ijiri and Potten, 1988, 1990).

*Tissue preparation*

The control and treated rats were sacrificed under sodium pentobarbitone (Nembutal) anaesthesia at 0 h (no treatment) and then at 0.5, 1, 2, 4, 6, 8, 12 and 24 h after irradiation. The small intestine and colorectum were removed, cleaned in sterile isotonic saline, cut and either fixed in 10% formal saline at 4°C overnight or flash frozen in liquid nitrogen for subsequent RNA extraction. Fixed samples were dehydrated in graded ethanols before paraffin embedding. Sections were cut at 4 μm for haematoxylin and eosin (H&E) staining and for immunohistochemical localisation of p53.

*Counts of apoptosis*

For analysis of intestinal crypts, the term 'crypt' was defined as the row of cells located on one side of a longitudinally sectioned crypt. Fifty crypts were selected for analysis from each of the four rats per group, using methods detailed in previous reports (Arai *et al.*, 1989, 1996). The incidence of cell death was quantified by counting the number of dead cells in the crypt. The distinctive morphological features of apoptosis, as described by Kerr *et al.* (1994, 1995) and Walker *et al.* (1988), were used to recognise apoptotic cells, and any doubtful cells were disregarded. Small clusters of dead cell fragments were assessed as originating from one cell and given a single count. We chose not to use the new method of end-labelling DNA strand breaks associated with apoptosis with biotin-labelled dUTP (Gavrieli *et al.*, 1992). Kerr *et al.* (1995) suggest that use of morphological criteria for identification of apoptotic cells is more reliable than the end-labelling technique. As well, previous work by us (Gobé *et al.*, 1995) and others (detailed in Grasl-Kraupp *et al.*, 1995) has shown that cell labelling occurs in early necrotic change and sometimes in mitotic nuclei, both of which are known to have DNA strand breaks, albeit transiently in mitotic cells, and that end-labelling results must be used with caution for assessment of levels of apoptosis.

*Preparation of cDNA encoding rat p53*

The rat cDNA probe was synthesised from 5 μg of total RNA extracted from the rat prostate after castration. A 769 basepair fragment of rat p53 cDNA (Soussi *et al.*, 1988) was amplified by PCR using the sense primer 5′-ATATTCTGCC-CACCACAGCGAC-3′ and antisense primer 5′-TTTCTTCC-TCTGTCCGACGGTC-3′. Reactions were carried out in a volume of 40 μl and consisted of 1 μl of rat cDNA, two primers (sense and antisense, 5 μM each), 250 μM deoxy-ribonucleotide triphosphate (dNTPs), 1× standard PCR buffer, 1.5 mM magnesium chloride and 1 unit of *Taq* polymerase (Promega Biotec, USA). The samples were overlaid with mineral oil and subjected to 30 cycles of amplification in a Perkin-Elmer thermal cycler. Before the first cycle, the samples were denatured at 94°C for 4 min. The standard temperature profile of the cycles was as follows: denaturation at 94°C for 45 s, annealing at 55°C for 45 s and extension at 72°C for 2 min. The last extension step was carried out at 72°C for 10 min.

*Extraction of RNA and Northern blot analysis*

Total RNA was extracted from a series of flash frozen tissue obtained from untreated and treated rats at sequential intervals after irradiation, using a routine guanidinium thiocyanate method. Total RNA (20 μg) from each time point was electrophoresed in a 1.2% formaldehyde–agarose gel and transferred by capillary blotting onto a Hybond N+membrane (Amersham International, Australia). The membranes were hybridised to rat p53 cDNA probe labelled with [$\alpha^{32}$P]dCTP by the random primer method (Sambrook *et al.*, 1989). Loading of RNA gels was monitored by levels of ethidium bromide stain in gels, and reprobing the membranes with [$\alpha$-$^{32}$P]-labelled probe for $\beta$-actin. Intensity of autoradiographic bands was measured by densitometry using an ImageQuant (Molecular Dynamics, USA). Level of p53 transcript was adjusted for variation in the intensity of $\beta$-actin (loading control).

*Immunohistochemical analysis of p53 protein*

Endogenous peroxidase in sections was blocked by treatment with 0.3% hydrogen peroxide in methanol for 15 min. The sections were heated at 100°C for 10 min using microwave antigen-retrieval treatment (Bankfalvi *et al.*, 1994). Slides were immunostained using a labelled streptavidin–biotin method. Rabbit p53 polyclonal antibody (Ab-7, Oncogene Science, USA), streptavidin–horseradish peroxidase (Vector Laboratories, USA), and diaminobenzidine chromogen (DAB) were used. Counterstaining was with haematoxylin. Cells positive for p53 were identified by brown staining nuclei, whereas p53-negative nuclei were pale blue. Negative control slides were treated with non-immune serum in place of the p53 antibody. Background staining of nuclei with DAB was not seen.

*Analysis of the distributions of apoptotic fragments and p53-positive cells*

The distribution of apoptotic fragments or p53-positive cells for each cell position was obtained. In order to compare the distribution of apoptotic fragments with that of p53-positive cells, three parameters were calculated that describe each distribution: (1) a measure of central tendency, the median cell position ($\chi_{med}$) and the mode cell position of the distribution; (2) a measure of the spread of the right half of the distribution (the standard deviation of the right half, $\sigma_r$); and (3) a similar measure of the spread of the left half of the distribution ($\sigma_l$). These were calculated as described by Ijiri and Potten (1983). Median values were plotted against time after treatment to obtain a regression line which could be extrapolated back to time zero ($t_0$). From these calculations, the median value of the target population susceptible to radiation-induced apoptosis at time $t=0$ could be assessed. These methods are described in detail by Potten *et al.* (1992).

*Statistical analysis*

Data were analysed by the Student's $t$-test and the Mann–Whitney $U$-test with a significance limit set at $P<0.05$ (Ichihara, 1990).

### Results

*Apoptotic indices*

Figure 1 is a temporal comparison of the mean apoptotic index (mean apoptotic cells per crypt) in small and large intestine after 2 Gy irradiation, for four animals at each time point. The data for rats sacrificed at 09.00 h without any treatment (baseline level) are shown as the 0 h value. The control level (0 h) for the small and large intestine was $0.04 \pm 0.01$ and $0.01 \pm 0.00$ apoptotic cells per crypt respectively, and these counts did not differ significantly from apoptotic counts in tissue from the control animals collected at each time point. After irradiation, an increased incidence of apoptotic cells from the control level was first observed at 2 h in the small intestine. At this time, the apoptotic index in the small intestine was significantly higher than that in the large intestine ($P<0.01$). Apoptotic indices peaked at 2–4 h in the small intestine compared with 6–8 h in the large intestine, indicating an earlier response to irradiation in the small intestine compared with the large intestine. The averages of the highest incidence of apoptotic cells were

**Figure 1** Temporal alterations of mean apoptotic indices (apoptotic index, number of apoptotic cells per 'crypt'. 'Crypt', row of cells on one side of a longitudinally sectioned intestinal crypt) in the rat small (□) and large (○) intestine after 2 Gy irradiation. Time 0 h represents control levels, which did not differ significantly from this level throughout the 24 h of experimentation. Error bars represent one s.d. from the mean result obtained from four rats per data point.

5.03 cells per crypt (range, 4.20–5.74) at 4 h in the small intestine, and 4.56 cells per crypt (range, 3.04–5.46) at 6 h in the large intestine. Apoptotic indices declined to low levels again by 24 h.

### *Expression of p53 mRNA*

Figure 2a shows representative Northern blots of control and irradiated tissue from the small and large intestine. p53 mRNA transcript was detected in control tissue of both small and large intestine (Figure 2a, lane 0). Densitometric analyses of p53 transcript levels corrected for loading levels (Figure 2b) indicated that p53 mRNA expression in the small and large intestine was reduced to approximately half of control levels by 8 h post-irradiation. In the large intestine, there was a trend for decreased expression that was not significantly different from the small intestine ($P>0.1$) when densitometry from all blots was averaged and analysed. An insignificant transient increase ($P>0.1$) in p53 mRNA over control levels at 4 h post-irradiation was seen. In summary, neither tissue showed a significant increase in p53 expression.

**Figure 2** (**a**), Representative autoradiographs from Northern blots showing expression of p53 and β-actin mRNA transcript levels in the small intestine (upper series) and large intestine (lower series). Total RNA (20 μg), extracted from the intestine at various time points in controls and after irradiation, was subjected to electrophoresis, transferred to Hybond N+ film, and hybridised with $^{32}$P-labelled probe for rat p53 or β-actin. (**b**), Graphic representation of densitometry that was used to analyse expression of p53 against the β-actin loading controls. After adjustment for equal loading in each lane, means of p53 mRNA were not significantly increased over control levels during observation. Error bars represent one s.d. from the mean.

### *Expression of p53 protein*

Expression of p53 protein in small and large intestine after irradiation was evaluated by immunohistochemistry (Figures 3a and b, Figure 4). In the control small and large intestine, few intestinal cells showed p53 antigenicity, and pattern and level of p53 immunolocalisation did not differ among the control sections for the 24 h of the experiment. Level of labelling in control sections is indicated at 0 h in Figure 4. Numerous positive cells were detected in the small and large intestine of the irradiated rats (Figure 3a and b respectively). p53 nuclear protein was found in viable epithelial cells of the small and large intestine. No positive staining was shown on the nuclear fragments of apoptotic cells. Occasional pericryptal fibroblasts or lymphocytes scattered in the lamina propria had positive staining (data not shown). Figure 4 details the frequency of p53 positivity, and shows that increases in p53 protein levels were detectable 30 min after irradiation, reaching a peak 4 h after irradiation, before reducing to normal levels by 24 h. In the small and large intestine, the time course of p53 positivity correlated well with the apoptotic index (detailed in Figure 1), although in the large intestine some discrepancy in the correlation was seen. In most experiments, 6- to 10-fold increases in p53-positive cells were noted after 2 Gy irradiation. Although the incidence of p53-positive cells in the large intestine was generally lower than that in the small intestine, there was no significant difference between corresponding values, except at 8 h after irradiation ($P<0.05$).

### *Distribution of apoptotic and p53-positive cells and their relationship*

The distribution of the apoptotic cells and p53-positive cells at each time point is shown in Figure 5. Distribution was often symmetrical, slightly skewed to the right in the small intestine, and strongly skewed to the right in the large intestine. It is notable that marked increase of p53 protein was observed 30 min after irradiation whereas the number of apoptotic cells was not increased at this time point. The peak positions of p53-positive cells were similar to those of apoptotic cells in both small and large intestine. These data are summarized in Table 1. The positions of target cells of the apoptotic process and p53-positive cells were $5.3\pm1.0$ and $5.2\pm0.9$ in the small intestine

**Figure 3** Immunohistochemical appearance of p53 protein expression in (**a**) the small intestine and (**b**) the large intestine of irradiated rats. Arrows indicate the p53-positive cells, which mainly distribute above the Paneth cell region in the small intestine and at the bottom of the crypt in the large intestine. Sections from control animals (not demonstrated) showed very little positivity.

**Figure 4** Temporal alterations of p53 protein expression (mean number of p53-positive cells per 'crypt') in the rat small (□) and large (○) intestine after irradiation. Note that there are up to 10-fold increases in the p53-positive cells observed at 2–4 h after irradiation, which parallel the incidence of apoptosis shown in Figure 1. Error bars represent s.d. from the mean.

respectively, and 5.5±0.8 and 6.0±1.4 in the large intestine respectively. There were no significant differences between target cell positions of the apoptotic cells and p53-positive cells. These findings demonstrated that in the small intestine the position of apoptotic and p53-positive cells and reported stem cell position (cell position 4–5) were coincident, whereas in the large intestine the stem cell population (cell position 1–2) was not coincident with target cell population after radiation treatment.

### Discussion

There are now several papers that demonstrate the importance of wild-type p53 in modulation of radiation-induced apoptosis in the gut (Clarke *et al.*, 1994; Merritt *et al.*, 1994). However, detailed information on the localisation of p53 expression is scant. In the present study, we have examined and compared the temporal and spatial alterations in p53 expression at various time points after irradiation in the rat small and large intestine.

Our results show increases of up to 10-fold over control levels in p53 protein in both small and large intestine. In comparison, p53 mRNA, measured by Northern blot, was not significantly altered. Northern blot methodology may be insufficiently sensitive to detect changes in mRNA expression when relatively small numbers of cells are involved. However, there are reports that have clearly demonstrated post-transcriptional regulation of p53 in DNA damaged cells (Kastan *et al.*, 1991; Lu and Lane, 1993). Thus, the disparity between p53 mRNA and protein expression found in the present study may be explained by involvement of post-transcriptional control mechanisms. Such post-transcriptional control is important. Rapid production of new protein would be possible from original non-damaged DNA, and production of new mRNA from potentially irradiation-damaged DNA template would become unnecessary or diminished.

Alterations in p53 protein levels were very rapid, peaking within 4 h in both the small and large intestine, and then reducing to approximately normal levels at 24 h. In comparison with our results using X-ray irradiation, increased p53 protein levels were reported to be detectable for at least 20 days after UV radiation (Fritsche *et al.*, 1993; Hall *et al.*, 1993; Lee and Bernstein, 1993; Lu and Lane, 1993; Nelson and Kastan, 1994). It is possible that the role of p53 protein in these two examples differs, or that the stimulus for p53 expression is important in conferring different types of p53 protein stability. Accumulation or retention of p53 protein may occur because of factors that increase protein stability, such as phosphorylation, protein–protein binding or oligomerisation of p53 (Kastan *et al.*, 1991).

Wild-type p53 appears to be crucial for DNA damage-induced apoptosis (Clarke *et al.*, 1993, 1994; Lowe *et al.*, 1993; Merritt *et al.*, 1994). Its increased expression is also necessary for induction of apoptosis in other tissues (Yonish-Rouach *et al.*, 1991; Shaw *et al.*, 1992; Ryan *et al.*, 1993). Our data have demonstrated that an increase in p53 protein expression preceeded the occurrence of apoptosis *in vivo*. Thus, p53 expression is temporally related to the induction of apoptosis in our model, although the spatial correlation was not so easily analysed. p53 protein was not detected in the fragmented nuclei of apoptotic cells and bodies, but rather on the nuclei of viable epithelial cells. In this case, the role for p53 may be cell cycle arrest and repair of damaged DNA in the surviving cell population (Ryan *et al.*, 1993; Yonish-Rouach *et al.*, 1993). Thus, p53-positive cells may consist of two kinds: cells undergoing apoptosis and surviving cells undergoing DNA repair.

Despite similarities in structure, pathophysiological differences between small and large intestine have been recognised for many years. We have recently demonstrated that clusterin mRNA is extensively expressed at the lower part of the small intestinal crypt, whereas it is diffusely observed in the large

**Figure 5** The distribution of mean percentage of apoptotic cells (□) and p53-positive cells (●) in the intestinal crypts of the untreated and irradiated rats. The distribution of apoptotic cells is closely correlated with that of p53-positive cells in both small intestine (**a**) and large intestine (**b**). Distribution of both parameters is mostly symmetrical, although a slight skew to the right is seen in the small intestine, and a more pronounced skew to the right in the large intestine, of occurrence of apoptosis over p53-positivity.

**Table I** Summary of data for the median ($\chi_{med}$) and mode values at the various time points (2–8 h after irradiation) in the small and large intestine

| *Cells examined* | *Small intestine* $\sigma_r$ | $\sigma_l$ | $\chi_{med}$ | *Position of mode at 2–8 h* | *Position of target cells at t=0* | *Large intestine* $\sigma_r$ | $\sigma_l$ | $\chi_{med}$ | *Position of mode at 2–8 h* | *Position of target cells at t=0* |
|---|---|---|---|---|---|---|---|---|---|---|
| Apoptotic cells | 5.5±0.4 | 3.7±0.3 | 6.9±0.6 | 2–8 | 5.3±1.0 | 5.7±0.4 | 3.5±0.4 | 5.9±0.5 | 1–3 | 5.5±0.8 |
| p53-positive cells | 5.3±1.2 | 2.0±0.3 | 4.5±0.5 | 3–5 | 5.2±0.9 | 5.3±1.1 | 2.4±0.8 | 4.1±1.2 | 2–3 | 6.0±1.4 |

intestine (Arai *et al*., 1996). Those results, results from the present study and reports from other workers (Ijiri and Potten, 1983; Ijiri, 1989) indicate that there is a more defined hierarchal formation in the small intestine than in the large intestine. Differences in the apoptotic rate among the various parts of the intestine have been reported (Ijiri, 1989; Ijiri and Potten, 1988, 1990). Apoptosis occurred more frequently in the small intestine than in the large intestine in response to irradiation. In contrast, the response to a different tissue insult, dimethyl hydrazine (DMH), gave inverse results. Although our results displayed no significant difference between the peak levels of the apoptotic indices in the small and large intestine, the response to irradiation in the small intestine appeared earlier than that seen in the large intestine. Our results do, however, indicate a close link between p53 expression and apoptosis, in that we found levels of apoptosis and p53 protein to be proportional. Thus, our data support a hypothesis that p53 function is essential for cellular response to radiation-induced DNA damage.

In a further attempt to define differences in response to irradiation in the small and large intestine with respect to differences in tumorigenicity, we compared published data of stem cell population positions with our results showing positions for cell death and p53 expression. In the small intestine, the target cells for radiation-induced apoptosis were calculated at cell position 5–6, being in accord with results from other investigators (Ijiri and Potten, 1983; Li *et al.*, 1992), and the target cell position for p53 expression was similarly placed. This position is also reported to house the stem cells of the small intestine (Potten, 1992). In the large intestine, the target cell population for apoptosis and p53 expression was again located at cell position 5–6. The stem cell in the large intestine is, however, postulated to be located at cell position 1–2 (Potten, 1992). Consequently, the occurrence of apoptotic cell deletion and p53 expression had shifted upwards from the stem cell population in the large intestine. Thus, radiation-damaged stem cells might be more effectively removed in the small intestine than in the large intestine. Radiation-damaged stem cells may tend to remain at the base of the large intestine and be related to later tumorigenesis.

In a recent substantial paper on p53 expression in spontaneous and radiation-induced apoptosis in the gut, Merritt *et al.* (1994) surmised that the colon (large intestine) lacked or had diminished function that would normally lead to apoptotic deletion of damaged cells. One significant difference between their results and ours, however, is that in our model expression of p53 protein in the large intestine was generally lower than that in the small intestine, whereas there was no significant difference between the incidence of apoptotic cells in the small and large intestine. The disparity between similar apoptotic indices in both intestines and weaker p53 accumulation in the large intestine indicates that an assumption that most p53-positive cells would undergo apoptosis is simplistic. Another aspect for consideration is the ability for DNA repair which may be weaker in the large intestine compared with the small intestine, such that in the large intestine the damaged cells are not only less effectively removed, but also less effectively repaired, leading to a higher incidence of tumorigenesis in this tissue.

**Acknowledgements**
The work presented in the paper was carried out in the Queensland Cancer Fund Research Laboratory at the Department of Pathology, University of Queensland. Financial support was from University of Queensland URG funds. The authors thank Professor JFR Kerr, recently retired Head of the Department of Pathology, University of Queensland, for his instructive suggestions. We are also grateful to Mr CM Winterford for preparing the photographs.

### References

ARAI T AND KINO I. (1989). Morphometrical and cell kinetic studies of normal human colorectum. Comparison between the proximal and the distal large intestine. *Acta Pathol. Jpn.*, **39**, 725–730.

ARAI T, KIDA Y, HARMON BV AND GOBE GC. (1996). Expression and localization of clusterin mRNA in the small and large intestine of irradiated rats: its relation with apoptosis. *Int. J. Radiat. Biol.* (in press).

BANKFALVI A, NAVABI H, BIER B, BOCKER W, JASANI B AND SCHMID KW. (1994). Wet autoclave pretreatment for antigen retrieval in diagnostic immunohistochemistry. *J. Pathol.*, **174**, 223–228.

BERGES RR, FURUYA Y, REMINGTON L, ENGLISH HF, JACKS T AND ISAACS JT. (1993). Cell proliferation, DNA repair, and p53 function are not required for programmed death of prostatic glandular cells induced by androgen ablation. *Proc. Natl Acad. Sci. USA*, **90**, 8910–8914.

CLARKE AR, PURDIE CA, HARRISON DJ, MORRIS RG, BIRD CC, HOOPER ML AND WYLLIE AH. (1993). Thymocyte apoptosis induced by p53-dependent and independent pathways. *Nature*, **362**, 849–852.

CLARKE AR, GLEDHILL S, HOOPER ML, BIRD CC AND WYLLIE AH. (1994). p53 dependence of early apoptotic and proliferation responses within the mouse intestinal epithelium following gamma-irradiation. *Oncogene*, **9**, 1767–1773.

DONEHOWER M, MONTOGOMERY CJ, HARVEY M, SLAGLE B, MCARTHUR M, BUTEL J AND BRADLEY A. (1992). Mice deficient for p53 are developmentally normal but susceptible to spontaneous tumours. *Nature*, **356**, 215–221.

FRITSCHE M, HAESSLER C AND BRANDNER G. (1993). Induction of nuclear accumulation of the tumor-suppressor protein p53 by DNA-damaging agents. *Oncogene*, **8**, 307–318.

GAVRIELI Y, SHERMAN Y AND BEN-SASSON SA. (1992). Identification of programmed cell death *in situ* via specific labeling of nuclear DNA fragmentation. *J. Cell Biol.*, **119**, 493–501.

GOBÉ GC, BUTTYAN R, WYBURN K, ETHERIDGE MR AND SMITH PJ. (1995). Clusterin expression and apoptosis in tissue remodelling associated with renal regeneration. *Kidney Int.*, **47**, 411–420.

GRASL-KRAUPP B, RUTTKAY-NEDECKY B, KOUDELKA H, BUKOWSKA K, BURSCH W AND SCHULTE-HERMANN R. (1995). *In situ* detection of fragmented DNA (TUNEL assay) fails to discriminate among apoptosis, necrosis, and autolytic cell death: a cautionary note. *Hepatology*, **21**, 1465–1468.

HALL PA, McKEE PH, MENAGE HP, DOVER R AND LANE DP. (1993). High levels of p53 protein in UV-irradiated normal human skin. *Oncogene*, **8**, 203–207.

ICHIHARA K. (1990). *Statistics for Bioscience: Practical Technique and Theory*. Nankodo: Tokyo.

IJIRI K. (1989). Apoptosis (cell death) induced in mouse bowel by 1,2-dimethylhydrazine, methylazomethanol acetate, and gamma-rays. *Cancer Res.*, **49**, 6342–6346.

IJIRI K AND POTTEN CS. (1983). Response of intestinal cells of differing topographical and hierarchial status to ten cytotoxic drugs and five sources of radiation. *Br. J. Cancer*, **47**, 175–185.

IJIRI K AND POTTEN CS. (1988). Circadian rhythms in the incidence of apoptotic cells and number of clonogenic cells in intestinal crypts after radiation using normal and reversed light condition. *Int. J. Radiat. Biol.*, **53**, 717–727.

IJIRI K AND POTTEN CS. (1990). The circadian rhythm for the number and intensity of radiation-induced apoptosis in the crypts of the small intestine. *Int. J. Radiat. Biol.*, **58**, 165–175.

KASTAN MB, ONYEKWERE O, SIDRANSKY D, VOGELSTEIN B AND CRAIG RW. (1991). Participation of p53 protein in the cellular response to DNA damage. *Cancer Res.* **52**, 6304–6311.

KERR JFR, WINTERFORD CM AND HARMON BV. (1994). Apoptosis. Its significance in cancer and cancer therapy. *Cancer*, **73**, 2013–2026.

KERR JFR, GOBÉ G, WINTERFORD C AND HARMON B. (1995). Anatomical methods in cell death. *Methods Cell Biol.*, **46**, 1–27.

LANE DP. (1992). p53, guardian of the genome. *Nature*, **358**, 15–16.

LEE JM AND BERNSTEIN A. (1993). p53 mutations increase resistance to ionizing radiation. *Proc. Natl Acad. Sci. USA*, **90**, 5742–5746.

LI YQ, FAN CY, O'CONNOR PJ, WINTON DJ AND POTTEN CS. (1992). Target cells for the cytotoxic effects of carcinogens in the murine small bowel. *Carcinogenesis*, **13**, 361–368.

LOWE SW, SCHMITT EM, SMITH SW, OSBORNE BA AND JACKS T. (1993). p53 is required for radiation-induced apoptosis in mouse thymocytes. *Nature*, **362**, 847–849.

LU X AND LANE DP. (1993). Differential induction of transcriptionally active p53 following UV or ionizing radiation: defects in chromosome instability syndromes? *Cell*, **75**, 765–778.

MERRITT AJ, POTTEN CS, KEMP CJ, HICKMAN JA, BALMAIN A, LANE DP AND HALL PA. (1994). The role of p53 in spontaneous and radiation-induced apoptosis in the gastrointestinal tract of normal and p53-deficient mice. *Cancer Res.*, **54**, 614–617.

NELSON WG AND KASTAN MB. (1994). DNA strand breaks: the DNA template alterations that trigger p53-dependent DNA damage response pathways. *Mol. Cell. Biol.*, **14**, 1815–1823.

POTTEN CS. (1992). The significance of spontaneous and induced apoptosis in the gastrointestinal tract of mice. *Cancer Metast. Rev.*, **11**, 179–195.

POTTEN CS, LI YQ, O'CONNOR PJ AND WINTON DJ. (1992). A possible explanation for the differential cancer incidence in the intestine, based on distribution of the cytotoxic effects of carcinogens in the murine large bowel. *Carcinogenesis*, **13**, 2305–2312.

RYAN JJ, DANISH R, GOTTLIEB CA AND CLARKE MF. (1993). Cell cycle analysis of p53-induced cell death in murine erythroleukemia cells. *Mol. Cell. Biol.*, **13**, 711–719.

SAMBROOK J, FRITSCH EF AND MANIATIS T. (1989). *Molecular Cloning. A Laboratory Manual.* 2nd ed. pp. 10.13–10.17. Cold Spring Harbor Laboratory Press: New York.

SHAW P, BOVEY R, TARDY S, SAHLI R, SORDAT B AND COSTA J. (1992). Induction of apoptosis by wild-type p53 in a human colon tumor-derived cell line. *Proc. Natl Acad. Sci. USA*, **89**, 4495–4499.

SOUSSI T, DE FROMENTEL CC, BREUGNOT C AND MAY E. (1988). Nucleotide sequence of a cDNA encoding the rat p53 nuclear oncoprotein. *Nucleic Acids Res.*, **16**, 11384.

WALKER NI, HARMON BV, GOBE GC AND KERR JFR. (1988). Patterns of cell death. *Methods Achiev. Exp. Pathol.*, **13**, 18–54.

YONISH-ROUACH E, RESNITZKY D, LOTEM J, SACHS L, KIMCHI A AND OREN M. (1991). Wild-type p53 induces apoptosis of myeloid leukaemic cells that is inhibited by interleukin-6. *Nature*, **352**, 345–347.

YONISH-ROUACH E, GRUNWALD D, WILDER S, KIMCHI A, MAY E, LAWRENCE JJ, MAY P AND OREN M. (1993). p53-mediated cell death: relationship to cell cycle control. *Mol. Cell. Biol.*, **13**, 1415–1423.

ZHANG X, COLOMBEL M, RAFFO A AND BUTTYAN R. (1994). Enhanced expression of p53 mRNA and protein in the regressing rat ventral prostate gland. *Biochem. Biophys. Res. Commun.*, **198**, 1189–1194.